# Circulating-Free DNA Analysis in Hepatocellular Carcinoma: A Promising Strategy to Improve Patients’ Management and Therapy Outcomes

**DOI:** 10.3390/ijms20215498

**Published:** 2019-11-05

**Authors:** Silvia Mezzalira, Elena De Mattia, Michela Guardascione, Chiara Dalle Fratte, Erika Cecchin, Giuseppe Toffoli

**Affiliations:** Clinical and Experimental Pharmacology, Centro di Riferimento Oncologico di Aviano (CRO) IRCCS, via Franco Gallini n. 2, 33081 Aviano (PN), Italy; silvia.mezzalira@cro.it (S.M.); michela.guardascione@cro.it (M.G.); chiara.dallefratte@cro.it (C.D.F.); ececchin@cro.it (E.C.); gtoffoli@cro.it (G.T.)

**Keywords:** hepatocellular carcinoma, circulating cell-free DNA, liquid biopsy, predictive/prognostic markers, disease monitoring, somatic mutational profile, methylation patterns, DNA fragmentation, next-generation sequencing

## Abstract

Hepatocellular carcinoma (HCC) is the sixth most common malignancy worldwide, representing the third leading cause of cancer-related deaths. HCC genetic characterization at the tumor level has been recently completed, highlighting how a number of genes are frequently mutated in this pathology. Actionable somatic mutations found in a HCC tumor may represent targets for innovative drugs as well as prognostic/predictive markers. Nonetheless, surgical or bioptic tissue is hardly accessible in HCC and a single tumor sample is poorly representative of the tumor genetic heterogeneity. In this context, analyzing the circulating cell-free DNA (ccfDNA) and its tumor-derived fraction (ctDNA) could represent a promising strategy of liquid biopsy. Recent data suggested that the fluctuation of the ccfDNA quantity in the plasma of HCC patients could anticipate the detection of tumor progression. The presence of somatic mutations in p53 signaling, Wnt/β-catenin, chromatin remodeling, response to oxidative stress and telomerase maintenance pathways can also be studied in ccfDNA bypassing the need to perform a tumor biopsy. The profiling of ccfDNA fragmentation and the methylation pattern could further improve the clinical management of HCC patients. Performing a dynamic monitoring in the course of systemic treatment with sorafenib or regorafenib is a possible way to provide insights into the resistance mechanism, and to identify predictive and prognostic genetic alterations, helping the clinicians in terms of treatment decision making. This review will discuss the most recent literature data about the use of ccfDNA to monitor and improve the treatment of HCC.

## 1. Introduction

Liver cancer is globally the fourth most common cause of cancer-related death and the sixth in terms of incidence [1]. Hepatocellular carcinoma (HCC) accounts for the majority of primary liver cancers. The most relevant HCC risk factor includes hepatitis B and C virus infections, alcoholism and metabolic syndrome [2]. The Barcelona Clinic Liver Cancer (BCLC) algorithm is the most widely applied staging system, which classifies patients as being in one of five stages and it provides treatment recommendations for each one [1]. Surgical resection, liver transplantation and local ablation are considered curative therapeutic practices for early-stage HCC, while other modalities, such as transarterial chemoembolization (TACE) and systemic therapy represent palliative options for the treatment of intermediate-advanced stage disease [3,4]. A number of small-molecule tyrosine kinase inhibitors such as sorafenib, regorafenib, cabozantinib and lenvatinib have demonstrated some survival benefits in advanced HCC and, more recently, promising data on the use of immune checkpoint inhibitors [5] are emerging.

Despite the aforementioned curative or palliative treatments, the prognosis of HCC patients is still poor. A number of studies have investigated the potential role of genetic markers to improve the management of HCC patients. The host genetic profile (i.e., genetic polymorphisms) was demonstrated to contribute to the individual predisposition to develop HCC (e.g., variants in gene encoding UDP-glucuronosyltransferase 1A [UGT1A], DNA repair enzymes, glutathione S-transferases, membrane transporters, cytochromes [CYPs]) [6,7] as well as to the prediction of the HCC therapy outcome (e.g., variants in gene encoding drug metabolic enzymes as CYPs and UGT1A1/A9, membrane transporters, vascular endothelial growth factor (VEGF)-dependent and -independent pathways related-proteins) [5]. Other studies focused instead on the somatic HCC profile, exploring the genetic mutational status of liver tumor tissue and reporting the tumor suppressor *tumor protein p53 (TP53)* and the WNT pathway oncogene *catenin beta 1 (CTNNB1)* as the most frequently mutated genes [8,9]. Those investigations have further permitted the definition of distinct HCC molecular subtypes, which are related to different clinical and histological features, patient prognosis and therapy outcome. For example, HCC tumors harboring oncogenic *PI3K-MTOR* mutations had worse outcomes on sorafenib treatment, while the presence of an activating WNT/β-catenin alterations was associated with innate resistant to immune checkpoint inhibitors [8]. Moreover, the identification of HCC patients with potentially druggable mutations (e.g., *MTOR* and *MET*) could open new therapeutic opportunity [8,9].

However, it should be considered that liver cancer is one of the most heterogeneous tumors, therefore a single biopsy hardly represents the high genetic heterogeneity of the entire tumor lesion [10]. In addition, the HCC diagnosis can be established mostly without an invasive biopsy, using combined radiological and biological (i.e., alpha-fetoprotein [AFP] level) criteria. The baseline somatic mutations profile is, therefore, commonly not assessable due to the lack of either bioptic or surgical tissue, as for late stage patients with HCC when only systemic treatments are recommended.

In this context liquid biopsy represents a great opportunity to perform a non-invasive analysis of tumor molecular alterations, since the circulating tumor (ctDNA), a variable fraction of total circulating cell-free DNA (ccfDNA), have been shown to carry genetic information consistent with tumor cells [11,12]. In particular, Labgaa et al., using a ultra-deep targeted sequencing, confirmed the tumoral origin of the *cis* mutations found in plasma, providing definitive evidence of the release of HCC-derived DNA fragments into the bloodstream [11]. In addition, characterizing the somatic profile through the ctDNA analysis could return important information regarding the tumor heterogeneity and its dynamic evolution over time [13,14]. Even if the studies published so far are still few in number, the up-to-date data have highlighted the great clinical potentiality of the quantitative and qualitative (i.e., genetic and methylation profiles) analyses of the circulating DNA to improve the HCC early diagnosis as well as the treatment of both early- and late-stage patients with HCC.

This review aims to critically report and discuss the literature data on the role of quantitative and qualitative analysis of the circulating DNA as a novel strategy to improve the treatment and management of patients with HCC.

## 2. Circulating-Free DNA Investigation in Oncology

The presence of circulating nucleic acids in human blood was firstly described by Mandel and Métais in 1948 [15] but only 29 years later were ccfDNA serum levels observed to be significantly higher in cancer patients with respect to healthy donors [16], paving the way for further investigations upon the clinical implementation of ccfDNA analysis. Only in 1994 did the detection of amplifiable *KRAS* mutated copies in the serum of patients suffering from pancreatic carcinoma prove the tumor origin of a fraction of total ccfDNA [17]. In this work, the authors selectively amplified *KRAS*-mutated alleles in ccfDNA of pancreatic carcinoma patients by means of allele-specific polymerase chain reaction (PCR) and confirmed their results by Sanger sequencing. They observed that the presence of amplifiable *KRAS*-mutated alleles was an exclusive feature of pancreatic tumor tissue with respect to healthy cells.

Nowadays, it is commonly accepted that the biological release of ccfDNA from both healthy and tumor cells mainly relies on a mixture of active and passive processes, including apoptosis, necrosis and exosome-mediated secretion. Cell-death mechanisms are indeed accompanied by macrophage-mediated debris elimination, eventually resulting in the DNA shedding into the systemic circulation [18]. As well as the primary tumor cells, even metastatic niches and circulating tumor cells (CTCs) help release the ccfDNA tumor fraction. ccfDNA is commonly detectable as a mixture of different length fragments, the vast majority of them spanning between 80 and 200 nucleotides. It is noteworthy that this measure agrees with the length of DNA wrapped around the nucleosome proteins, suggesting that during the DNA cleavage process the nucleosome and chromatosome structures protect DNA from the nucleases’ activity. Consistent with this observation, ccfDNA was reported to circulate also embedded in nucleosomes and chromatosomes structures [19]. ccfDNA undergoes physiologic elimination processes mainly mediated by the liver and kidneys and its half-life is estimated as ranging from 20 minutes to two hours. The central role that ccfDNA could play as a real-time biomarker in cancer research is evident, considering its fast clearance.

Since ccfDNA in cancer patients is composed of variable fractions of tumor-derived DNA (i.e., ctDNA) and healthy cells-derived germline DNA (gDNA), the ability of selectively detect and quantify the tumor fraction is relevant to assess tumor genetic characteristics. Indeed, ctDNA displays the same molecular features of the origin tissue with the great advantage of including the relative contribution of different tumor clones and metastasis, thus describing well the tumor molecular heterogeneity. Clinical utilities of ccfDNA analysis comprise a wide range of applications as early diagnosis, treatment response evaluation, identification of acquired resistance or relapse and minimal residual disease (MRD) monitoring.

Although increased ccfDNA levels are commonly associated with the tumor presence and the association between ccfDNA concentrations and oncological disease was largely demonstrated [20], the mere ccfDNA quantification could provide only limited information upon tumor presence. Indeed, a number of non-oncological conditions could lead to a sharp increase of ccfDNA in body fluids. Therefore, the identification of molecular markers of oncological origin (as somatic mutations) could refine the analysis of ccfDNA and provide evidence of its derivation from a tumor tissue. Even if this approach could not clarify the type of tumor tissue from which the ctDNA originated it could at least allow it to be discriminated from DNA deriving from healthy tissues. However the detection of a somatic mutation as a hallmark of cancer in ccfDNA is limited by the low level of free DNA in plasma. To date, traditional sequencing approaches, mainly represented by Sanger sequencing, do not permit the achievement of the desired sensitivity, thus representing over the past years the main limiting step in ccfDNA analysis. In the course of the last 10 years, the development of digital-polymerase chain reaction (PCR)-based techniques has boosted the specificity and sensitivity of the analytical approaches in ccfDNA research. Nowadays, the spread of digital next-generation sequencing (NGS) techniques permitted analysis of genetic variants in very diluted DNA samples such as those extracted from the plasmatic biological matrix [21] and the identification of poorly represented tumor DNA sequences, thereby enabling the discrimination of low-abundance somatic mutations.

Aiming at developing a cancer screening test, Cohen et al. analyzed both genetic and protein biomarkers in plasma samples of 1005 patients, sequencing a narrow panel of 16 cancer-relevant genes. The test provided a 33–98% (median 70%) of sensitivity in detecting cancer cases according to the tumor type and the respective rate of DNA shedding in the plasma and a false positive rate of 0.9% in healthy donors [22]. Notably, higher-stage tumors release a higher quantity of ctDNA in the plasma and are consequently easier to be studied through the analysis of ccfDNA whereas the identification of the early-stage tumors remains a still-unmet clinical need.

In the framework of cancer treatment one of the main challenges in clinical practice is the early assessment of treatment response. In metastatic breast cancer patients, higher ctDNA levels were demonstrated to be correlated with a worst overall survival (OS) and ctDNA detection was also able to provide an earlier evaluation of treatment response in 53% of analyzed women compared with other biomarkers, such as CA15-3 and CTCs enumeration [23]. More recently, with the aim of creating a clinical predictive model for the early evaluation of immune check-point inhibitors (i.e., anti-programmed cell death protein-1 [PD-1], anti-programmed death-ligand 1 [PD-L1], anti-cytotoxic T-lymphocyte antigen 4 [CTLA4]) response, Jensen et al. carried out a low-coverage whole genome sequencing of ccfDNA in 56 patients receiving immunotherapies demonstrating that the genome-instability (GIN) score they developed can be relevant for discriminating clinical response from progression, differentiating progression from pseudo-progression and for identifying the hyper-progressive disease [24].

With particular regard to targeted therapies, the early identification of secondary acquired resistance is another field that deserves to be better investigated by means of ccfDNA analysis. Indeed, the clinical response to anti-epidermal growth factor receptor (EGFR) tyrosine kinase inhibitors in non-small-cell lung carcinoma patients was estimated with a sensitivity of 96% by evaluating the absence of tumor-specific T790M *EGFR* mutation, commonly associated with resistance against anti-EGFR first generation compounds. Moreover, ccfDNA sequencing revealed the presence of *EGFR* mutations responsible for acquired resistance against anti-EGFR third-generation compounds [25].

Interrogating ctDNA to assess MRD in cancer patients represents a powerful strategy for relapse prediction. Tie et al. demonstrated, considering a prospective population of stage II colorectal cancer patients, the correlation between post-surgery ctDNA concentrations and tumor recurrence. Notably, patients with ctDNA detectable after surgery showed a 10-fold higher risk of developing recurrence when compared with patients without ctDNA detectable after surgery [26].

## 3. Liquid Biopsy to Improve Hepatocellular Carcinoma (HCC) Therapeutic Management

**Figure 1:**
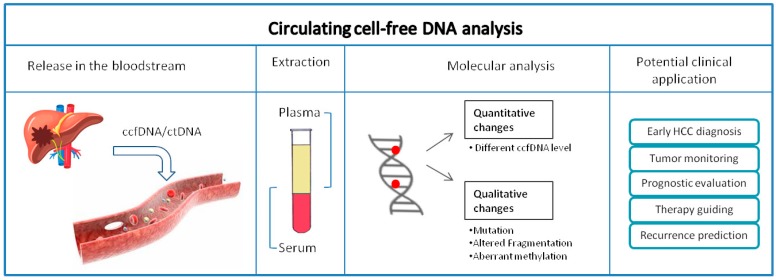
Schematic overview of circulating cell-free DNA analysis and its potential clinical application in hepatocellular carcinoma (HCC) setting. Circulating cell-free DNA (ccfDNA) can be released in the bloodstream from a variety of different cells under physiological and pathophysiological conditions. In cancer patients, a fraction of ccfDNA comprises circulating tumor DNA (ctDNA). ccfDNA can enter systemic circulation where can be isolated from serum or plasma. ccfDNA can undergo both quantitative (i.e., monitoring of changes in the ccfDNA concentration) and qualitative (somatic mutational profile, altered fragmentation and aberrant methylation pattern) analysis. The evaluation of the ccfDNA and its tumoral fraction, ctDNA, can improve the management of HCC patients permitting an early diagnosis, a better tumor monitoring (i.e., recurrence prediction, supervision of the dynamic tumor evolution) and an improved therapy outcome prediction that finally help clinicians in the treatment decision making.

In the context of HCC, ccfDNA has been studied with the aim of defining both an early diagnostic marker of tumor disease evolution and a non-invasive marker of therapeutic outcome and patients’ prognosis (Figure 1). Different methodological approaches have been adopted focusing either on ccfDNA level in plasma or on the somatic mutational profiling and methylation features of ccfDNA of tumor origin [27]. The quantity of ccfDNA in the bloodstream in cancer patients was demonstrated to be higher as compared to healthy or non-cancer patients. A qualitative analysis of the ccfDNA permits researchers to identify the tumor specific point mutations, the alteration in the integrity of the DNA, the aberrant methylation patterns or allelic imbalance. More recently the dimension of circulating DNA fragments has been related to its origin, creating new opportunities for a specific detection of circulating DNA deriving from the tumor tissue [28,29,30,31,32]. This information can be helpful in monitoring the disease evolution driving different treatment strategies. Few prospective studies have investigated ccfDNA level and its characteristics to predict clinical outcomes and to help clinical decision making in the context of HCC management.

### 3.1. Circulating Cell-Free DNA Level and HCC Clinical Outcome

The first studies investigating the relationship between ccfDNA and HCC treatment outcome were aimed at evaluating the variation in the amount of ccfDNA in patients’ plasma (Table 1). The rationale behind this approach arose from the evidence that the total ccfDNA is partly of tumor derivation and, thus, an increase in ccfDNA plasma concentrations could be related to the tumor′s presence or dimension. Many investigations have focused on the relationship between ccfDNA levels and the presence of cancer by analyzing healthy and cancer patients, in order to establish a cut-off value able to discriminate the two groups. Furthermore, in addition to the risk-assessment studies, prognostic analyses on ccfDNA have been carried out to point out its relationship with stage/aggressiveness of the disease or treatment response.

Initially, most of the studies focused on the diagnostic potentiality of measuring ccfDNA levels. Case-control studies were designed and carried out to identify the difference in the plasma ccfDNA level between healthy volunteers and patients with HCC, usually defining a study-dependant cut off [33,34,35,36]. In particular, in the study of Ren et al. [33] a significant difference in plasma ccfDNA level was highlighted by comparing 79 patients with HCC to 20 healthy volunteers. In this investigation, the cut off concentration discriminating patients with HCC from healthy subjects, was set at 36.6 ng/mL. No difference was found instead comparing patients with HCC to 20 patients with liver cirrhosis. Tokuhisa and colleagues [35] investigated the differences in ccfDNA levels in 96 post-surgical Japanese patients with HCC affected by hepatitis C virus (HCV), and comparing them to 100 patients with HCV unaffected by HCC. In this study ccfDNA levels were significantly higher in patients with HCV affected by HCC than in patients with HCV unaffected by HCC. In this case ccfDNA had a mean concentration of 115.9 ± 98.3 ng/mL in patients with HCC-HCV, and 34.4 ± 40.4 ng/mL in patients with HCV. By contrast with the work of Ren et al. [33], the analysis was performed using the serum instead of plasma for quantifying the ccfDNA through a quantitative real-time PCR-based evaluation of the glutathione S-transferase P 1 (*GSTP1*) gene amplification. In 2012 Huang et al. [34], using the same real-time PCR-based technique targeting the ß-actin gene, analysed plasma ccfDNA level in 72 post-surgical Chinese patients with HCC, comparing them to 37 subjects with benign liver disease, and 41 healthy volunteers. By setting the concentration of 173 ng/mL as the study-defined cut off, the analysis reported significantly higher concentration of ccfDNA in patients with HCC as compared to non-tumor patients, either healthy or benign liver disease controls, with a median of 173.9 and 46 ng/mL respectively. The study of Piciocchi and colleagues [36] aimed to identify the importance of the ccfDNA quantification as a diagnostic tool in HCC by real-time PCR amplification targeting the telomerase reverse transcriptase (*hTERT*) gene. To that end, 66 patients with HCC, 35 with cirrhosis and 41 with advanced HCV-related chronic hepatitis were enrolled. Plasma ccfDNA levels were measured showing a mean concentration of 9.5 ± 2.5 ng/μL in patients with HCC, 5.1 ± 1.3 ng/μL in patients affected by cirrhosis and 1.6 ± 0.23 ng/μL in patients with chronic hepatitis. Despite the variation in the ccfDNA levels among the three groups, the differences were not statistically significant using a cut off value of 1 ng/μL of extracted DNA calculated by Receiver Operating Characteristic (ROC) analysis. Another study to be mentioned is the work of Oh et al. [37], reporting that ccfDNA concentration in 151 patients with HCC treated with sorafenib 400 mg twice a day was significantly higher than in 14 healthy volunteers (0.71 ng/μL vs 0.34 ng/μL, *p* < 0.0001).

In the aforementioned investigations, the authors took into consideration also the predictive/prognostic value of the ccfDNA concentration measured in the plasma or serum samples of the patients with HCC. Ren et al. [33], assessed the prognostic role of ccfDNA level in defining the post-surgical clinical outcome in terms of 3-years disease-free survival (DFS) and OS. The results of the study showed that a high DNA concentration (cut off of 36.6 ng/mL) in patients’ plasma was an independent factor associated with a shorter DFS and OS (*p* = 0.004 and *p* < 0.001, respectively). Three-year DFS rates for “high plasma DNA” and “low plasma DNA” group were 22% and 47%, (*p* = 0.008), whereas 3-year OS rates were 24% and 61%, respectively (*p* < 0.001). Similarly, the study of Tokuhisa and colleagues [35] assessed the relationship between post-operative serum ccfDNA level and post-surgical patient DFS, recurrence risk and OS within 18 months of follow up. After establishing a serum ccfDNA cut off level of 117.8 ng/mL, the authors reported that a higher serum ccfDNA level was significantly associated with shorter OS (*p* = 0.0017) while no remarkable association was detected for DFS. Then, the authors investigated the relationship between post-operative ccfDNA level and the risk of developing early intrahepatic recurrence (within 1 year of surgery). Even though the serum ccfDNA level was reported to be significantly higher in patients experiencing early intrahepatic recurrence than in those without intrahepatic recurrence (*p* = 0.0017), it was not an independent risk factor associated with early intrahepatic recurrence. On the contrary, ccfDNA level emerged as an independent prognostic factor for extrahepatic recurrence, with patients showing high serum ccfDNA level displaying a 4.5-fold increased risk of developing recurrence in distant organs. The clinical endpoint of OS after HCC surgery has been evaluated also in the study of Huang and colleagues [34]. In agreement with the results of Tokuhisa et al. [35], higher ccfDNA concentrations were associated with shorter OS but in this case, the difference did not reach statistical significance (*p* = 0.071). In the same work, a dynamic evaluation of ccfDNA level in plasma was performed in 20 out of 72 patients with HCC through the analysis of a second plasma sample collected one to six months after surgery for monitoring level changes after treatment. A decreased concentration in the plasma ccfDNA was observed in the second plasma sample in comparison to the concentration measured at the time of surgery (median value: 42 ng/mL versus 173 ng/mL), suggesting that the ccfDNA level variation could identify the efficacy of the surgical resection. Piciocchi and colleagues [36] used a similar approach. They split the study population into two groups with high level or low level of ccfDNA (cut off: 2 ng/μL). The analysis pointed out that patients with ccfDNA level below the cut off showed a median survival of 37 months, 13 months longer than patients with level above the cut off. The results were confirmed when restricting the analysis to patients with a viral-related etiology (hepatitis B virus [HBV] and/or HCV) or with only HCV- related liver disease: median survival was shorter in patients with ccfDNA above the cut off (24 months vs. 29 months, respectively). Disease control rate was analysed by the work of Oh et al. [37] who highlighted a significant association (*p* = 0.003) between higher ccfDNA level and worse disease control rate, using a cut off value of 0.82 ng/µL to divide patients into ccfDNA-high group and ccfDNA-low group. In addition, the ccfDNA-high group had a worse time to progression (TTP) (2.2 vs. 4.1 months; hazard ratio (HR)  =  1.71; *p* = 0.002) and OS (4.1 vs. 14.8 months; HR  =  3.50; *p* < 0.0001) than the ccfDNA-low group. In the multivariable analyses, the ccfDNA remained an independent prognostic factor for OS (*p* < 0.0001).

The work of Park et al. [38], focused instead on patients with HCC treated with radiotherapy-chemotherapy (RT-CT). Between June and April 2011, 55 Korean patients with HCC who had received a RT-CT treatment were recruited and included in two different cohorts according to different RT-CT schedules: the first cohort of 34 subjects underwent conventionally fractionated RT (CFRT) with concomitant 5-fluorouracil/cisplatin chemotherapy and the second cohort of 21 patients received stereotactic body radiation therapy (SBRT). Pre- and post-RT plasma samples were collected from patients and the ccfDNA level was evaluated spectrophotometrically using an ultraviolet-visible spectrophotometer. Using a cut off value of 37.25 ng/mL ccfDNA level in plasma, patients were divided into post-RT “low ccfDNA” and “high ccfDNA” groups. Treatment response data, as a radiographic response to an irradiated lesion, suggested a significant better treatment response in patients with low ccfDNA level post-RT as compared to subjects with high level (*p* = 0.017). OS and progression-free survival (PFS) were not significantly related to ccfDNA level, while intrahepatic failure-free (IHFF), and local control (LC) rates were lower in “high ccfDNA” respect to “low ccfDNA” group. A post-RT subgroup analysis was also performed stratifying patients according to the treatment arm (CFRT versus SBRT): in this case IHFF rates were not significantly different, while LC rate was better in low DNA respect to high DNA group, both for SBRT (*p* = 0.041) and for CFRT arm (*p* = 0.046).

Although many scientific papers reported a diagnostic and prognostic/predictive capacity of the ccfDNA level, some weaknesses remain, limiting a potential clinical application of the ccfDNA quantitative analysis. Firstly there is no consensus about the proper cut off value to apply for the discrimination of high and low ccfDNA concentration; this cut off is strongly laboratory-dependent resulting in a poor reproducibility of the data. Moreover, the concentration has been evaluated in different matrixes: plasma or serum. In fact, both plasma and serum have frequently been used in this type of analysis, but many studies reported how the concentration in serum samples is significantly higher than the concentration in matched plasma samples, depending on the differential level of white blood cell lysis [39]. Consequently, it follows that serum is not suitable for ccfDNA level monitoring. This approach is also limited by the lack of specificity of the ccfDNA level parameter for the DNA of tumor origin, making the measure strongly dependent from the sample quality and the processing method. As already noted, ccfDNA has several possible origins, ranging from cells apoptosis to necrosis and the total levels rise in a number of disorders including serious infection, inflammatory condition [40], and myocardial infarction [41]. For these reasons, some results are still controversial and, although a potential clinical value of ctDNA has been reported by many authors, the quantitative analysis as a diagnostic/prognostic test remains a debated issue. Therefore, over the years, thanks to the technology advancement in sequencing, the quantitative evaluation has been frequently supported by a qualitative analysis, able to detect specific tumor DNA features in plasma.

### 3.2. Circulating Cell-Free DNA Genetic Profiling and HCC Clinical Outcome

In addition to quantitative changes analysis, a qualitative changes evaluation, in terms of sequence variation and methylation pattern of circulating DNA, was performed by several research groups. The detection of tumor-specific genetic mutations from ctDNA, technically difficult to perform in the past, is now feasible thanks to the development of specialized digital techniques with high analytical sensitivity. Available studies, listed in Table 2, used either a targeted approach or a whole-genome and exome sequencing approach (WGS, WES) to identify new variants impacting the therapeutic outcome of patients with HCC or the disease progression itself. Sequence alterations detected globally to date in the ctDNA of patients with HCC were in genes involved in the maintenance of telomeres (i.e., *TERT*), in the tumor suppression (i.e., *TP53*), and in the regulation of cell growth and adhesion (i.e., *CTNNB1*) [1,42,43,44]. The revision of the current literature has brought out several papers focusing on the possible role of ctDNA sequence variations as biomarkers for disease recurrence in different therapeutic settings, at various stages of the disease (Table 2).

In the context of early stages of HCC, where surgery and TACE are the major treatment options, three studies [44,45] analysed the prognostic role of the genetic variants in ctDNA. In the work of Liao et al. [44], between December 2013 and August 2014, 41 Chinese patients with primary HCC were enrolled in order to assess the relationship between tumor-associated mutations and post-surgery recurrence-free survival (RFS). Using a NGS-based method, a targeted panel of hot-spot regions in the three most relevant genes associated to HCC (i.e., *TERT*, *TP53* and *CTNNB1*) were analysed in plasma ccfDNA and matched tumor samples. Somatic mutations were consistently detected in both ccfDNA and tumor samples in 8 of 41 patients (19.5%): 2 patients revealed *TERT* mutations, 4 *CTNNB1* mutations and 2 *TP53* mutations. The median RFS for patients with tumor-associated mutations detected in ccfDNA was 89 days compared with 365 days for patients with no plasma mutation. These data revealed a major probability to relapse related to the presence of somatic mutations in ctDNA of patients after surgical treatment. The same clinical endpoint (i.e., recurrence probability) in relation with the somatic ctDNA mutational profile, was also investigated by a recent work of Cai et al. [45]. This study demonstrated that the comprehensive ctDNA mutation profiles could accurately and better estimate patients′ prognostic risk and detect tumor occurrence in advance respect to traditional strategies as imaging (Computed Tomography/Magnetic Resonance Imaging) and serum protein biomarkers. Particularly, the study was performed on 34 long-term follow-up patients with HCC who received surgical resection followed by other adjuvant therapies during follow-up. Primary tumor tissue as well as ctDNA derived from plasma samples collected at preoperative, postoperative, and multiple follow-up time points were characterized for somatic single-nucleotide variants (SNVs) and copy-number variants (CNVs) by targeted deep sequencing and low-coverage WGS. Besides confirming the consistency of the somatic profile between pre-surgery plasma derived ctDNA and matched primary tumor tissue, the study highlighted that patients with high SNV/CNV fractions in pre-operative ctDNA presented worse clinic-pathological features as well as shorter RFS and OS respect to low SNV/CNV group. Moreover, during follow-up, dynamic change in the SNV and CNV profile was significantly correlated to patients′ tumor burden consistent with imaging results. A model integrating the comprehensive ctDNA mutation profiles was also developed and was found to predict tumor occurrence in advance of imaging for an average of 4.6 months, and with a superior performance than serum biomarkers; the same model was also shown to detect MRD and predict patients′ prognostic outcomes for both RFS and OS. ctDNA integration with the serum biomarker des-gamma-carboxy prothrombin further improves the predictive performance of the model. These data highlighted the potentiality of the ctDNA-based strategy as a useful and non-invasive tool for dynamically monitoring HCC progression, which could be further combined with the traditional methods providing a better evaluation of tumor burden and supporting the treatment decision making.

On the other hand, when considering HCC at an advanced stage, it must be considered that only systemic pharmacological treatments are used. The study of by Oh et al. [37] was aimed at identifying ccfDNA-based biomarkers for the prediction of treatment outcome in HCC patients treated with sorafenib. To this aim, the authors investigated the overall ccfDNA copy number alteration (CNA) in 151 patients and vascular endothelial growth factor A (*VEGFA*) gene amplification in a subset of 41 patients before sorafenib treatment by using low-coverage WGS. Pre-sorafenib patients with HCC had higher level of *VEGF* gene amplification and CNAs in plasma as compared to 14 healthy controls. On the basis of the CNA detected in plasma, the authors computed a genomic instability score, named ”Iscore”, to evaluate the degree of chromosomal instability in HCC patients. Notably, a higher I-score, which reflects a higher rate of genome instability, resulted significantly associated to a shorter TTP, a lower disease control rate (DCR) and a worse OS. The association between *VEGFA* copy number variation and the clinical end-points analysed was not significant.

The reported studies depicted an emerging important role of pre-treatment analysis of tumor-specific gene alteration: both with specific point analysis and genome-wide analysis it is possible to predict clinical outcomes to different treatments, including surgery or systemic treatments.

### 3.3. Circulating Cell-Free DNA Methylation Profiling and HCC Clinical Outcome

DNA methylation is an hereditary epigenetic sign consisting of a methyl group covalent transfer to the C-5 position of the cytosine DNA ring mediated by *DNA methyltransferase* (DNMT) [46]. In mammals, over 98% of DNA methylation occurs in a context of CpG dinucleotide in somatic cells and 2% methylation occurs in a non-CpG context in embryonic stem cells (ESC). The CpG islands are regions abundant in regulatory regions and promoters of eukaryotic genes characterised by the presence of a cytosine followed by a guanine nucleotide. DNA methylation is essential for normal development, playing an important role in key processes and, if dysregulated, it contributes to diseases such as cancer. The methylation of specific gene promoter is a well-known mechanism for transcriptional repression [47] which results in gene silencing [48]. On this basis, it emerges that aberrant methylation in a cancer-related gene promoter represents a tumor-specific event firstly associated to tumorigenesis [49]. Hypermethylation and hypomethylation typically occur in the CpG islands of the gene promoter region and, due to specific gene inactivation, had been involved in the development and progression of cancer through different processes [50]. Consequently, the detection of alteration in the DNA methylation could be potentially useful for the prediction, diagnosis and prognosis of patients with HCC [47]. The methylation pattern analysis includes some advantages over somatic mutation detection such as higher sensitivity, a dynamic range and the presence of a large amount of target regions. In the clinical practice, nowadays, a few methylation markers are validated, such as *septin 9* (*SEPT9*) in colorectal cancer [51], and several groups have reported that the analysis of circulating methylated tumor suppressor genes could be used for the non-invasive detection of human tumors, including HCC [43,52,53,54,55].

Concerning HCC, data related to alteration in DNA methylation of different genes including *TP53*, tumor protein p16 (*p16*), Adenomatous Polyposis Coli (APC) regulator of WNT signalling pathway, serine peptidase inhibitor, kunitz type 2 (*SPINT2*), secreted frizzled related protein 1 (*SFRP1*), tissue factor pathway inhibitor 2 (*TFPI2*), *GSTP1* and Ras association domain family 1 isoform A (*RASSF1A*) have been reported, and these patterns were often associated with cancer initiation and progression [27]. However, only limited results are available regarding the impact of circulating DNA methylation on HCC therapy outcomes (Table 3).

In 2008 Chan et al. [55] analysed quantitative changes in circulating methylated marker as a diagnostic and/or prognostic biomarker in HCC setting. Sixty-three post-surgical patients with HCC, 63 age- and sex-matched chronic HBV carriers, and 50 healthy volunteers were enrolled in the study. Three blood samples were collected from each patient affected by HCC (at the time of diagnosis, and at 1 month and 1 year after the surgery resection). Hypermethylation of the tumor suppressor gene *RASSF1* in plasma was evaluated as a potential diagnostic or prognostic marker for HCC. Hypermethylated *RASSF1A* was detectable by real-time PCR in the serum of 93% of the HCC patients before surgery, 58% of HBV carriers, and 8% of the healthy volunteers. Of 59 patients with detectable methylated *RASSF1A* in the serum at diagnosis, 45 patients (76%) showed a reduction in the concentration of circulating methylated *RASSF1A,* one month after tumor resection. In addition, patients with a concentration of serum-methylated *RASSF1A* greater than the cut off value of 1 × 106 copies at diagnosis, showed a significantly poorer disease-free survival than the patients with lower concentrations [55].

Xu and colleagues [49] analysed a panel of 401 candidate methylation markers detectable in a consistent manner in the tumor tissue of patients with HCC and matched ccfDNA from plasma. This set of markers, analysed in the ccfDNA from a group of 715 patients with HCC and 560 healthy individuals permitted the development of a diagnostic score including 10 methylation markers. Adopting a similar approach, they developed a prognostic score comparing the methylation markers distribution within patients affected by HCC with different prognosis. An 8-marker score was validated in an independent group of patients and was demonstrated to discriminate patients with significantly different risk of death (Log-Rank *p* < 0.0001).

The hypermethylation of *RASSF1A*, evaluated in combination with another three genes (*APC, GSTP1* and *SFRP1*), was analysed by the group coordinated by Huang et al. [34]. The analysis was carried out on 72 patients with HCC, 37 with benign liver disease, and 41 healthy volunteers by quantitative methylation-sensitive restriction enzyme digestion PCR (MSRE-PCR). This study highlighted that the level of methylation of *RASSF1A* in plasma was an independent prognostic factor for OS. Similar to the results obtained by Chan et al., patients with elevated plasma methylation levels of *RASSF1A* or *APC* showed poorer OS than subjects with low levels. No correlation has emerged between *GSTP1* and *SFRP1* methylation level and OS.

Another work [53] focused on the analysis of *p15* and *p16* methylation patterns in tumor DNA. Surgical hepatic specimens were taken from 25 patients with HCC along with matched plasma/serum samples taken during the 14 months following surgery. In addition, plasma/serum samples of 20 healthy individuals and 35 chronic hepatitis or cirrhosis controls were collected as control samples, to analyse the association between aberrant gene methylation and the development of recurrence or metastases. *p15* and *p16* were detected to be methylated in 92% of surgical specimens and in 74% of the plasma/serum samples patients. Association with the clinical endpoint indicated that 75% (9 of 12) of patients affected by HCC with concurrent *p15* and *p16* methylation (as measured by tissue analysis) developed liver recurrence or lung metastasis during a median follow-up time of 14 months post-surgery, as compared to no patient (0 of 12) in non-methylated tumors. These data suggest how ccfDNA methylation profile analysis could be an alternative diagnostic and prognostic method for non-invasive HCC disease monitoring.

### 3.4. ccfDNA Selection by Length in HCC

Since the discovery of detectable tumor-derived DNA in the blood of cancer patients, many efforts have been made to selectively quantify it with always increased sensitivity. To overtake detection limits arose from the identification of tumor-specific mutations, which could lead to underestimating the ctDNA amount, researchers have struggled to detect common features shared by the overall ctDNA molecules in the bloodstream. The principle whereby cancer cells die as a consequence of several mechanisms, including necrosis, apoptosis and autophagy [56,57], whereas normal cells commonly die through apoptosis, has been leveraged to investigate a possible difference in ctDNA dimension and fragmentation pattern that could represent ctDNA in its entirety. Thus, the selective quantification of ccfDNA molecules based on their length, has led to the definition of a ccfDNA integrity (cfDI), defined as the ratio of long over short fragments. In the first studies evaluating the relative contribution of long and short ccfDNA fragments by means of selective PCR-based amplification of different size amplicons, the cfDI was found to be significantly higher in cancer patients than in healthy subjects [58,59]. Furthermore the cfDI rate was observed to significantly increase in late-stage cancer patients, when compared with early stage cancers and it was commonly associated with a poor prognosis [60]. To date, in HCC few studies investigating the size profile of ccfDNA showed contradictory results. However, the use of different ccfDNA source (plasma, serum) and of different analytical approaches hampers the achievement of universal consent.

Chen et al. [28], estimated the serum ccfDNA integrity in a cohort of 80 HBV-related patients with HCC and compared it with that reported in HBV patients without HCC (*n* = 80) and in healthy subjects (*n* = 50). qPCR was used to selectively amplify short (100 bp) and long (400 bp) ccfDNA fragments in the ß-actin gene, while its integrity was defined as the ratio between the concentrations of the long and short fragments. They found out that patients with HCC show significantly higher ccfDNA integrity rate than HBV patients without HCC (*p* < 0.001) and healthy subjects (*p* < 0.001). By a ROC curve analysis, a serum DNA integrity cut off of 0.36 and 0.34 was selected, thus allowing the discrimination of patients with HCC from HBV patients (sensitivity 78%, specificity 85%) and from healthy subjects (sensitivity 86%, specificity 91%), respectively [28].

In a cohort of 69 patients suffering from liver malignancies, including 53 HCC and 16 non-HCC liver cancer patients, Huang et al. [29] investigated the plasma ccfDNA integrity by selectively amplifying short (115 bp) and long (247 bp) ALU sequences by means of qPCR. Integrity of ccfDNA was calculated as the ratio between ALU 247 and ALU 115. In contrast with Chen et al. they observed that the integrity of ccfDNA in cancer patients was significantly lower than in patients with benign liver disease (*p* = 0.0167) and healthy controls (*p* = 0.0025). Notably, no difference in ccfDNA integrity between patients with HCC and without HCC was observed (*p* = 0.7356), suggesting that this biomarker is not specific for HCC diagnosis but that could be employed to monitor the disease, as proposed by the authors. However, by using a ROC curve, the authors estimated a ccfDNA integrity rate of 0.400 as the best cut off permitting to achieve a sensitivity of 43.3% and a specificity of 100%, in discriminating patients with HCC from healthy volunteers [28].

Jiang et al. [30] exploited CNA affecting chromosomes 1 and 8, which are peculiar to HCC, to identify the tumor-derived DNA fraction in plasma of 90 patients with HCC and to assess the differences in size of ccfDNA fragments of tumor-derived and non-tumor derived DNA. They observed that increasing tumor-derived DNA fraction correlated positively with a reduction in DNA fragments length (*p* < 0.001 for fragments below 150 bp), whereas the reduction of tumor-derived DNA correlated negatively with an increase in DNA fragment length (*p* < 0.001 for fragments above 180 bp) [30]. Moreover, by sequencing ccfDNA at a genome scale with a low-coverage approach, they further discovered that tumor-derived DNA in patients with HCC exhibits characteristic end-coordinates, sustaining the theory about a non-random DNA cleavage during apoptotic process [32]. By analyzing the size distribution of DNA molecules displaying tumor characteristic end-coordinates in the 90 patients with HCC, they confirmed that tumor-derived DNA bearing a specific molecular signature in its extremity is shorter than DNA without tumor-related end-coordinates [31]. These results suggest that ctDNA carries a plethora of characteristics from the tissue of origin, of which only a small fraction was investigated, and that the footprint of ctDNA must be sought by combining different analytical approaches.

## 4. Potential Issues Related to the Clinical Application of ccfDNA Analysis

Even if the evaluation of ccfDNA as a potential surrogate marker for tumor molecular profiling is surely a promising strategy to improve the management of patients with HCC, a number of methodological issues are still outstanding and currently limit the clinical use of ccfDNA analysis.

Firstly, a few reliable parameters can be evaluated up to date to discriminate with high specificity between circulating DNA deriving from tumor cells or from healthy tissues. To date, only the identification of tumor somatic mutation permits unequivocal association with the tumor DNA. However, the sensitivity of this approach is hampered by the limited number of hot-spot regions that can be analysed in parallel and by the variable amount of ctDNA shed in the bloodstream, which could be very poor especially in the early-stage cancers. Thus, ancillary strategies have been investigated in order to improve the specificity and sensitivity of ctDNA detection. Among them there are the evaluation of the size of ccfDNA in plasma, which seems to be related to the tissue of origins and to be significantly shorter for tumor-deriving ccfDNA [28,30] or the in silico analysis of the fragmentation pattern of ctDNA, which exhibits preferred end-coordinates [31].

Taking into account the analysis of tumor-circulating DNA as an early diagnostic test for tumor diagnosis two major hurdles should be considered. The first is the detection limit (i.e., sensitivity and signal stability) of the currently available technologies that affects the success of a ctDNA analysis. This represents a problem especially when the analysis of the ctDNA is performed in patients with an early-stage disease and, therefore, with lower amounts of circulating DNA. On the other hand, the ctDNA as a liquid biopsy tool seems closer to its clinical application in the subset of patients with advanced liver disease presenting higher ctDNA concentration. Overcoming the technical challenges posed by the low fraction of ctDNA within ccfDNA in early-stage HCC would be crucial for expanding the clinical application of this marker in all the HCC settings.

The second issue is related to the test specificity in a potential diagnostic setting, especially when focusing on circulating tumor DNA somatic mutations, since different types of tumors could be characterized by mutations in the same genes as *TP53*, *KRAS*, or *BRAF*. An early diagnosis based on the detection of tumor mutation in ctDNA would leave many unresolved issues regarding the primary location of the disease. In this sense, the analysis of epigenetic markers could probably be more useful since epigenetic biomarkers, as the highly tissue-specific DNA methylation profile, have been suggested to represent a good strategy to determine the tissue origin of ccfDNA although further targeted studies are necessary to better clarify this issue.

In order to overtake these present limitations hampering the clinical application of the liquid biopsy in clinical practice, the use of a panel of multiple biomarkers could represent a promising approach. With this in mind, the analysis of tumor-associated mutations in ccfDNA by means of high-sensitivity techniques (NGS) coupled with the identification of CNA in ccfDNA using ultra-low coverage WGS, the methylation profiling of ccfDNA and the concomitant quantification of protein biomarkers could increase the sensitivity and the specificity of liquid biopsy in HCC with specific regard to the early stages of the disease. To achieve this goal, combined efforts by multiple research groups in a wide population cohort will be required.

In view of a potential introduction in the clinical practice of the ccfDNA analysis, another target to be reached is the standardization of a series of technical and methodological parameters related to sample management, genetic platforms, and data analysis. At present, the high heterogeneity of all these aspects hampers the interpretation of available studies published so far. Concerning pre-analytical aspects, the isolation of the plasma or serum from the blood, the procedures for ccfDNA extraction and quantification, the condition for the ccfDNA storage as well as the samples’ collection timing crucial for the data interpretation should be considered. The experimental design, the method of ccfDNA detection and the choice of the analytical platform (e.g., NGS or digital PCR) as well as the raw data processing, data management and data quality control method (e.g., molecular barcoding, in silico error suppression), are all additional crucial factors requiring standardization. Most of the current studies employed different technical approaches, platforms and assays that result in diverse sensitivity and specificity. As far as genotyping methods are concerned, most of the studies used either digital PCR or NGS. While the first technology offers a high-sensitivity analysis of a limited number of single candidate genetic loci (e.g., useful for a quantitative monitoring of ctDNA through the assessment of tumor-associated genetic mutations), the NGS-based approaches allow researchers to interrogate the entire sequence of a large panel of multiple genes permitting also the identification of novel or epigenetic alteration (e.g., useful for obtaining tumor mutation profiling or monitoring tumor clonal evolution). However, even if the NGS-based approaches seem to offer more potentiality, their optimization is still more cost-, time-, and resource-consuming compared to digital PCR; hence, the requirement of an ultra-deep, high-coverage NGS-methods could represent another technical and cost barrier to the routine implementation of ctDNA-based analysis in clinical practice. Besides the methodological procedures, it should also be important to control for all the environmental and clinical confounding factors related to the patient, because it could affect the ccfDNA concentration (e.g., change in the therapeutic protocol, concomitant drug administration, concomitant disease, modification in the dietary and life-style habits) to produce comparable results.

## 5. Conclusions

Despite the recent advances in the clinical management of patients with HCC, including the introduction of improved surgical techniques and novel targeted therapy as well as the development of a comprehensive treatment plan, the 5-year survival rate has not significantly increased.

The analysis of ctDNA is increasingly included in translational and clinical trials, especially to detect MRD or to monitor the response to pharmacological treatments and tumor clonal evolution and may represent a powerful tool to help unmet challenges in the screening and management of HCC.

The most consistent results to date concern the longitudinal analysis of the plasma level of ccfDNA, as a dynamic real time marker of disease burden allowing researchers to anticipate the diagnosis of disease recurrence or tumor progression in patients receiving either a systemic or a local treatment. Only recently and thanks to the advancement of digital-sequencing technology some studies highlighted how ccfDNA could also be used as a source for somatic mutation detection. Although only few and preliminary studies approached this topic, this sequencing approach will probably refine in the future the study of ccfDNA in plasma, allowing researchers to more clearly identify the tumor DNA fraction in the plasma. In this sense, a very promising approach has been recently proposed focusing on the sizing of ccfDNA that seems to be closely related to the tissue of origin of the circulating DNA. This approach is very interesting since it would enable as never before researchers to identify the derivation of ccfDNA in a very sensitive and specific way.

Further dedicated multicenter, large, well-designed and long-term studies will be set up for overcoming the current conceptual and analytical limits of the use of ctDNA in clinical practice and to fully understand the real potentialities of this new technology. Moreover, with the increasing burden of data derived from liquid biopsy analyses together with the greater knowledge of the molecular and clinical complexity of the HCC disease, novel research methods based on big data management and artificial intelligence will be taken into consideration.

## Figures and Tables

**Table 1:** Level of circulating cell-free DNA (ccfDNA) and therapy outcome in hepatocellular carcinoma (HCC) patients.

Study Population	Therapy	Analyte	Measure Methods	Serum/ Plasma	Clinical Endpoint	Main Finding	Ref
HCC patients (*n* = 79) Cirrhotic patients (*n* = 20) Healthy volunteers (*n* = 20) (Chinese)	Surgery	ccfDNA level	Ultraviolet transilluminator system	Plasma	3 years DFS, OS, tumor feature	Compared with the healthy volunteers (17.6 ± 9.5 ng/mL), a significant higher ccfDNA level was found in the patients with HCC (47.1 ± 43.7 ng/mL, *p* = 0.000) or with liver cirrhosis (30.0 ± 13.3 ng/mL, *p* = 0.002). ccfDNA was closely associated with tumor size (*p* = 0.008) and TNM stage (*p* = 0.040), negatively associated with the 3-DFS (*p* = 0.017) and OS (*p* = 0.001).	[33]
HCC patients (*n* = 72) Cirrhotic/chronic hepatitis patients (*n* = 37) Healthy volunteers (*n* = 41) (Chinese)	Surgery	ccfDNA level	Quantitative RT-PCR	Plasma	OS, tumor feature	Plasma DNA concentrations were significantly higher in HCC patients compared with those in healthy controls or in benign controls (median 173 ng/mL, 9 ng/mL; 46 ng/mL, Mann–Whitney U test, *p* < 0.01). ccfDNA levels were positively associated with tumor size (*p* = 0.012), and were significantly elevated in HCC patients with intrahepatic spreading or vascular invasion (*p* = 0.035). Patients with ccfDNA level higher than the cut off value (173 ng/mL) (*n* = 29) showed a no-significant shorter OS respect those with low ccfDNA level (*p* = 0.017).	[34]
HCV-related HCC patients (*n* = 87) HCV carriers (*n* = 100) (Japanese)	Surgery	GSTP1	Quantitative RT-PCR	Serum	OS, DFS, tumor feature	Serum ccfDNA levels were significantly higher in HCC patients than in HCV carriers without HCC. ccfDNA levels were not associated with any clinic-pathologic factors. Patients with ccfDNA level higher than the cut off value (117.8 ng/mL) (*n* = 29) showed a significantly shorter OS compared to those with low ccfDNA level (*n* = 58) (*p* = 0.017) Serum ccfDNA levels were not associated with DFS.	[35]
HCC patients (*n* = 55) (Korean)	CFRT (*n* = 34) −45 Gy/25 fractions (*n* = 6) −45 Gy/25 fractions + chemotherapy (5-FU, cisplatin) (*n* = 28). SBRT (*n* = 21) (60 Gy/4 fractions)	ccfDNA level	Ultraviolet-visible spectrophotometry (Nanodrop2000)	Plasma	Tumor feature, response, OS, PF, IHFF, LC.	Pre-RT and post-RT ccfDNA level were measured. Patients were divided in high DNA (HDNA) and low DNA (LDNA) level group, both for pre-RT and post-RT using cut-off value of 33.65 ng/mL and 37.25 ng/mL respectively. Pre-RT HDNA group tended to have larger tumors (*p* = 0.017). Mean pre-RT ccfDNA values were similar for both groups (responders vs. non responders: 39.5 vs. 39.6 ng/mL, *p* = 0.988), but were significantly different post-RT (responders vs. non responders: 35.9 vs. 56.1 ng/mL, *p* = 0.002). Treatment response was significantly better in the post-RT LDNA group than the post-RT HDNA group (81.8% vs. 47.8%, *p* = 0.017). OS and PF rates were not significantly associated with different post-RT ccfDNA level. Tumor response, IHFF and LC rates were significantly better in the post-RT LDNA group r compared to the HDNA group (*p* = 0.017, *p* = 0.035, and *p* = 0.006, respectively).	[38]
Advance/metastatic HCC patients (*n* = 151) Healthy volunteers (*n* = 14) (Korean)	Systemic therapy (sorafenib 400 mg twice daily)	ccfDNA level		Plasma	DCR TTP OS	ccfDNA concentration in HCC patients was significantly higher than in healthy volunteers (0.71 ng/μL vs 0.34 ng/μL, *p* < 0.0001). Regarding HCC patients, DCR was significantly lower in ccfDNA-high group than in ccfDNA-low group using a cut off value of 0.82 ng/μL (*p* = 0.003). Moreover, the ccfDNA-high group had worse TTP (2.2 vs. 4.1 months; HR = 1.71; *p* = 0.002) and OS (4.1 vs. 14.8 months; HR = 3.50; *p* < 0.0001) than the ccfDNA-low group. In the multivariable analyses, the ccfDNA remained an independent prognostic factor for OS (*p* < 0.0001).	[37]
Viral-related (i.e., HBV or HCV) advanced chronic hepatitis or cirrhotic HCC patients (*n* = 66) Cirrhotic patients (*n* = 35) Advanced HCV-related chronic hepatitis patients (*n* = 41) (Italian)	Not available	h-TERT	Quantitative RT-PCR	Plasma	OS	HCC patients ccfDNA concentration was higher than in the other groups, but not statistically significant (*p* = 0.02, one-way analysis of variance (ANOVA)). Patients with ccfDNA level below the cut off value (2ng/μL) showed an improvement in OS compared with patients with ccfDNA level above the cut off value (37 months vs 24 months, *p* = 0.03).	[36]

Abbreviations: 3 years DFS, 3-years disease-free survival; 5-FU, 5-fluorouracil; ccfDNA, cell-free DNA; CFRT, conventionally fractionated radiation therapy; DCR, disease control rate; GSTP1, glutathione S-transferase *p* 1; HBV, hepatitis B virus; HCC, hepatocellular carcinoma; HCV, hepatitis C virus; HDNA, high DNA; hTERT, human telomerase reverse transcriptase; IHFF, intrahepatic failure-free; LC, local control; LDNA, low-DNA; OS, overall survival; PF, progression-free; RT, radiation therapy; quantitative RT-PCR, quantitative real-time polymerase chain reaction; SBRT, stereotactic body radiation therapy; TNM, tumor-nodes-metastasis; TTP, time to progression.

**Table 2:** Genetic profile of circulating tumor DNA (ctDNA) and therapy outcome in hepatocellular carcinoma (HCC) patients.

Study Population	Therapy	Analyte	Measure Methods	Serum/Plasma	Clinical Endpoint	Main Finding	Ref
Early-stage HCC patients (*n* = 41) Healthy volunteers (*n* = 6) (Chinese)	Surgery	TERT, CTNNB1, TP53	MiSeq sequencing	Plasma	RFS	Eight of the 40 patients successfully analyzed presented tumor-associated mutations. Patients with mutations in ctDNA were more likely to relapse (89 days for patients with somatic mutation vs. 365 days for patients without somatic mutation, *p* < 0.001).	[44]
Long-term follow-up patients with HCC (*n* = 34) (Chinese)	Surgery plus other adjuvant therapies (e.g., TACE radiofrequency ablation, target therapy) during follow-up.	Tumor somatic SNVs and CNVs	Target sequencing and low-coverage WGS	Plasma	MRD RFS OS	All plasma samples before surgery showed somatic genetic variations profile resembling corresponding primary matched tumor tissues. Patient groups with high SNV/CNV fractions evaluated in preoperative plasma samples have significantly poorer RFS (SNV, *p* = 0.0019; CNV, *p* = 0.001) and OS (SNV, *p* = 0.003; CNV, *p* = 0.0067) when compared with low SNV/CNV fractions. Moreover, increasing SNV fraction and CNV fraction were related to increasing tumor size, presence of microvascular invasion, and more severe tumor differentiation. During follow-up, SNVs and CNVs dynamically changed correlating to patients′ tumor burden. A model based on acquired SNV information was developed and was shown to accurately assess patients′ tumor burden with high consistence compared with imaging results. This model could discover tumor occurrence in advance of imaging for an average of 4.6 months, and showed superior performance than serum biomarkers (i.e., AFP, AFP-L3%, DCP). The model could also precisely detect MRD in advance and predict patients′ RFS (*p* = 0.001) and OS (*p* = 0.001). Furthermore combining ctDNA with DCP could increase the sensitivity for MRD detection, providing better prognostic value for both RFS (log-rank, *p* < 0.0001) and OS (log-rank, *p* < 0.0001) than ctDNA or DCP alone	[45]
Advance/metastatic HCC patients (*n* = 151) (Korean)	Systemic therapy (sorafenib 400 mg twice daily)	CNA, EIF2C1 (VEGFA-to-EIF2C1 ratio)	NextSeq 500 illumina low depth whole-genome sequencing	Plasma	DCR, TTP, OS	DCR and TTP did not significantly differ between the VEGFA-high and VEGFA-low group (*p* = 0.309 and *p* = 0.781). OS was reported shorter in VEGFA-high group than in VEGFA-low group even if it was not statistically significant (7.5 and 12.8 months respectively, *p* = 0.180). An high i-score, used as a CNA variation alternative, was correlated with worse DCR, TTP and OS (*p* = 0.0003, *p* < 0.0001 and *p* < 0.0001).	[37]

Abbreviations: AFP, alpha-fetoprotein; AFP-L3%, alpha-fetoprotein-L3; CNA, copy number alteration; CNV, copy number variation; CTNNB1, Catenin Beta 1; DCR, disease control rate; DCP, des-gamma-carboxy prothrombin; EIF2C1, eukaryotic translation initiation factor 2C1; HCC, hepatocellular carcinoma; MRD, minimal residual disease; OS, overall survival; RFS, recurrence-free survival; SNV, single-nucleotide variants; TACE, transcatheter arterial chemoembolization; TERT, telomerase reverse transcriptase; TP53, tumor protein p53; TTP, time to progression; VEGFA, vascular endothelial growth factor A; WGS, whole-genome sequencing.

**Table 3:** Methylation profile of circulating tumor DNA (ctDNA) and therapy outcome in hepatocellular carcinoma (HCC) patients.

Study Population	Therapy	Analyte	Measure Methods	Serum/plasma	Clinical Endpoint	Main Finding	Ref
HCC patients (*n* = 25) Hepatitis/cirrhotic patients (*n* = 35) Healthy volunteers (*n* = 20) (Chinese)	Surgery	p15, p16	MSP, southern blot	Serum/plasma	Recurrence	Methylation of p15 and p16 were found in 92% of tumor sample and in 74% plasma/serum sample. During a median follow-up time of 14 months post-surgery, 75% (9 of 12) of HCC patients with concurrent p15 and p16 methylation in tumors, 3 of 12 with only p16 methylation and 1 of 12 with only p15 methylation developed liver recurrence or lung metastasis. No p15 or p16 methylation were found in healthy or in hepatitis/cirrhotic non-HCC patients.	[53]
HCC patients (*n* = 63) HBV patients (*n* = 63) Healthy volunteers (*n* = 50) (Chinese)	Surgery	RASSF1A	MSP	Serum	DFS	Hypermethylated RASSF1A was detected in 93% of HCC patients, 58% of HBV carriers, and 8% of the healthy volunteers. The median RASSF1A concentrations for the HCC patients and HBV carriers were 7.70 × 105 copies/L and 1.18 × 105 copies/L, respectively. Patients with higher RASSF1A concentrations at diagnosis or 1 year after tumor resection showed poorer DFS (*p* < 0.01).	[55]
Training data set: HCC patients (*n* = 680) Validation data set: HCC patients (*n* = 369) (Chinese)	Heterogeneous treatment	401 genes (training data set) 8 genes (validation data set)	Target bisulfite sequencing-illumina sequencing	Plasma	OS	A prognostic prediction model was constructed with an independent 8-genes panel and a combined prognosis score system was generated (cp-score). Patients were divided in high- and low-risk groups, based on cp-score. OS was longer in the low risk group than in high risk group (cut off value −0.24).	[49]
HCC patients (*n* = 72) Cirrhotic patients (*n* = 25) Chronic inactive hepatitis (*n* = 12) Healthy volunteers (*n* = 41) (Chinese)	Not available	APC, GSTP1, RASSF1A, SFRP1	MSRE-qPCR	Plasma	OS	Elevated plasma methylation levels of APC or RASSF1A was associated with significantly poorer OS (Log-rank test, *p* < 0.05), while no significant association was found between plasma GSTP1 or SFRP1 methylation and OS (Log-rank test, *p* > 0.05). Cox multivariate analysis demonstrated that the methylation level of RASSF1A in plasma was an independent prognostic factor for OS (HR = 3.262, 95% CI:1.476–7.209. *p* = 0.003).	[34]

Abbreviations: APC, APC regulator of WNT signaling pathway; CI, confidence interval; DFS, disease free survival; GSTP1, Glutathione S-transferase P 1; HBV, hepatitis B virus; HCC, hepatocellular carcinoma; HR, hazard ratio; MSP, methylation-specific polymerase chain reaction; MSRE-qPCR, methylation-sensitive enzymes-based quantitative PCR; OS, overall survival; p15, or CDKN2B cyclin-dependent kinase 4 inhibitor B; p16, or CDKN2A cyclin-dependent kinase 4 inhibitor A; RASSF1A, Ras association domain family 1 isoform A; SFRP1, secreted frizzled related protein 1.

## Abbreviations

AFP	Alpha-fetoprotein
APC	Adenomatous Polyposis Coli
BCLC	Barcelona Clinic Liver Cancer
CNA	Copy number alteration
cfDI	Cell-free DNA integrity
ccfDNA	Circulating Cell-free DNA
CFRT	Conventionally fractionated RT
CNVs	Copy-number variants
CTCs	Circulating tumor cells
ctDNA	Circulating tumor DNA
CTLA4	Cytotoxic T-lymphocyte antigen 4
CTNNB1	Catenin beta 1
DCR	Disease control rate
DFS	Disease free survival
DNMT	DNA methyltransferase
EGFR	Epidermal growth factor receptor
ESC	Embryonic stem cells
GIN	Genome-instability
GSTP1	Glutathione S-transferase P1
HBV	Hepatitis B virus
HCC	Hepatocellular carcinoma
HCV	Hepatitis C virus
hTERT	Human telomerase reverse transcriptase
IHFF	Intrahepatic failure-free
KRAS	KRAS proto-oncogene GTPase
LC	Local control
MRD	Minimal residual disease
MSRE-PCR	Methylation-sensitive restriction enzyme digestion PCR
NGS	Next-generation sequencing
OS	Overall survival
PCR	Polymerase chain reaction
p16	Protein p16
PD-1	Programmed cell death protein-1
PD-L1	Programmed death-ligand 1
PFS	Progression-free survival
RASSF1A	Ras association domain family 1 isoform A
RT-CT	Radio-chemotherapy
SBRT	Stereotactic body radiation therapy;
SEPT9	Septin 9
SFRP1	Secreted frizzled related protein 1
SNVs	Single-nucleotide variants
SPINT2	Serine peptidase inhibitor, kunitz type 2
TACE	Transarterial chemoembolization
TFPI2	Tissue factor pathway inhibitor 2
TMB	Tumor mutational burden
TP53	Tumor protein p53
TTP	Time to progression
VEGFA	Vascular endothelial growth factor A
WES	Whole-exome sequencing
WGS	Whole-genome sequencing
